# Fault Diagnosis of Motor Bearings Based on a One-Dimensional Fusion Neural Network

**DOI:** 10.3390/s19010122

**Published:** 2019-01-02

**Authors:** Xianzhong Jian, Wenlong Li, Xuguang Guo, Ruzhi Wang

**Affiliations:** 1 School of Optical-Electrical and Computer Engineering, University of Shanghai for Science and Technology, Shanghai 200093, China; xgguo@usst.edu.cn; 2 School of Mechanical Engineering, University of Shanghai for Science and Technology, Shanghai 200093, China; WenLoG_Li@163.com; 3 School of Materials Science and Engineering, Beijing University of Technology, Beijing 100020, China; wrz@bjut.edu.cn

**Keywords:** motor bearings, fault diagnosis, deep learning, one-dimensional fusion neural network, D-S evidence theory

## Abstract

Deep learning has been an important topic in fault diagnosis of motor bearings, which can avoid the need for extensive domain expertise and cumbersome artificial feature extraction. However, existing neural networks have low fault recognition rates and low adaptability under variable load conditions. In order to solve these problems, we propose a one-dimensional fusion neural network (OFNN), which combines Adaptive one-dimensional Convolution Neural Networks with Wide Kernel (ACNN-W) and Dempster-Shafer (D-S) evidence theory. Firstly, the original vibration time-domain signals of a motor bearing acquired by two sensors are resampled. Then, four frameworks of ACNN-W optimized by RMSprop are utilized to learn features adaptively and pre-classify them with Softmax classifiers. Finally, the D-S evidence theory is used to comprehensively determine the class vector output by the Softmax classifiers to achieve fault detection of the bearing. The proposed method adapts to different load conditions by incorporating complementary or conflicting evidences from different sensors through experiments on the Case Western Reserve University (CWRU) motor bearing database. Experimental results show that the proposed method can effectively enhance the cross-domain adaptive ability of the model and has a better diagnostic accuracy than other existing experimental methods.

## 1. Introduction

Bearings are one of the most important mechanical components in rotating machinery. According to statistics, 40% of motor failures are bearing failures [1]. In order to reduce maintenance costs and prevent harmful or even damaging consequences of faults and failures, we need to find and replace fault bearings early. There are many ways to detect motor bearing faults, such as fault diagnosis through stray flux analysis [2], Park’s vector method (PVA) [3], instantaneous power factor (IPF) monitoring [4] and so on. Among them, using the acceleration of the motor housing to determine the existence of these faults is the most accurate method, which has become a very well-developed field in recent years [5]. Real time bearing vibration signal fault detection methods, including traditional methods and deep learning methods, have become a hot topic recently [6].

The traditional methods require feature extraction of the bearing vibration signal, dimension reduction, and classification. Feature extraction is mainly for signals in the time-domain, frequency domain and time-frequency domain. Traditional time-domain techniques extract features from time-domain vibration signals, including peak-to-peak, root mean square, crest factor, kurtosis, and pulse factor. The frequency domain technique extracts the corresponding features by transforming the time-domain signal into the frequency domain using Fast Fourier Transform (FFT) or other methods. At present, many time-frequency techniques such as atomic decomposition based wavelet transform (WT), empirical mode decomposition (EMD) and so on [7] have been applied in mechanical fault diagnosis. The descending dimension algorithms mainly include principal component analysis (PCA) [8] and independent component analysis (ICA) [9]. The classification methods include k-nearest neighbor (KNN) [10], support vector machine (SVM) [11] and so on. However, selecting the best features from the original feature set is a blind and subjective task. Moreover, due to the large number of monitoring points of electromechanical equipment and the high sampling frequency of the sensor, the detection system will obtain a large amount of data [12]. Correspondingly, there are two major challenges for fault diagnosis: (1), the amount of data is large and needs to be detected automatically; (2), the data types are diverse, and the features are difficult to extract. In recent years, deep learning has been developed rapidly. Since CNN has the ability to detect faults by learning optimal filters, it can directly extract and learn the best features from the original signals and it has been applied to behavior recognition [13], classification of ECG signals [14], speech recognition [15,16] and so on. CNN is superior to traditional methods not only in terms of accuracy, but also in terms of speed, and another key feature of CNN is its adaptive design. Based on these advantages, researchers have tried to apply deep learning to bearing fault diagnosis [17,18].

In [19], the time-frequency diagram of the rolling bearing signal is extracted as the input of the two-dimensional CNN, and the fault diagnosis is realized under the same network structure. Jia [20] proposed an FFT-DNN fault diagnosis method, which uses the preprocessed FFT spectrum image as the input of the DNN. Generally speaking, a two-dimensional image is equivalent to one surface, and a one-dimensional vibration signal is more like a line. We believe that the main reason for the application of CNN with two-dimensional convolution structure in image analysis is due to the two-dimensional spatial correlation in the image, but most bearing fault diagnosis measurement data are only related to one-dimensional time. If we directly convert it into a two-dimensional form, the spatial correlation in the original sequence will be destroyed and the information related to the failure may be lost [21].

Furthermore, bearings are usually operated with different loads. Under these different loads, problems such as different periods and phases of vibration signals, different amplitudes, and large waveform differences between different fault depths bring great challenges for bearing fault diagnosis [22]. Zhang [23] proposed the WDCNN algorithm for bearing fault type recognition and fault size assessment. It handles raw noisy signals and diagnoses bearing faults under different workloads. However, it still has a problem of over-fitting, which causes the network to not recognize the fault under variable load conditions. In addition, due to the complexity of the acquired vibration signals, or even the imbalance between different fault samples [24], the limitations of the individual model are highlighted [25]. At present, the deep learning model mainly focuses on the study of individual models, and there are also problems such as weak diagnostic generalization. The new technology of ensemble learning can combine multiple learners through some voting-like combination strategies to get better results [26]. In order to further improve the fault diagnosis ability, we propose a One-Dimensional Fusion Neural Network (OFNN).

We propose The One-Dimensional Fusion Neural Network combined D-S evidence theory with Adaptive one-dimensional Convolution Neural Networks with Wide Kernel (ACNN-W). Diagnostic models based on a single source of information may lead to misdiagnosis, and multi-sensor information sources can be used to obtain more reliable fault diagnosis through D-S evidence fusion. In recent years, D-S evidence theory and its variants have been widely used in multi-sensor data fusion, decision analysis, fault detection, and other industrial fields [27]. Most of the input signals for current deep learning fault diagnosis come from a single sensor. However, different sensors have different adaptability and anti-interference ability, which may result in failure to provide reliable information in a harsh working environment, so it makes sense to develop a learning model that takes advantage of deep learning and ensemble learning. Therefore, the proposed method uses D-S evidence theory to ensemble deep neural networks with multiple sensor information to construct a data fusion model for bearing fault diagnosis. The proposed fusion model can combine multiple uncertain evidences and provide fusion results by combining consensus information and exclusion information. It has the following characteristics:

(1) Compared with the machine learning-based damage assessment method proposed by the traditional method, the proposed method can adaptively extract features directly from the original vibration signal without manual feature extraction. Traditional machine learning damage detection methods use hand-crafted features that are not only sub-optimal, but also have high computational complexity.
(2) The ACNN-W can effectively suppress over-fitting and improve the generalization performance of the network.
(3) In order to overcome the limitations of individual deep learning models, reduce the impact of random initialization of neural networks, and make full use of information in different fields, we use D-S evidence theory to make comprehensive decisions on OFNN.

The remainder of this paper is as follows: Section 2 introduces the related work. Section 3 presents our method. Section 4 illustrates the evaluation of the ACNN-W model and optimizer and learning rate selection. Experiments in Section 5 expound the evaluation and analysis of the OFNN network. Section 6 is our conclusion.

## 2. Related Work

The method of classifying based on artificial extraction can be used in bearing fault diagnosis, but the method relies on manually acquired prior knowledge and cumbersome manual feature extraction, and the final accuracy is greatly affected by the selected features. Therefore, in this paper, we adopt a deep learning scheme to combine feature extraction and feature classification into one step. Some present methods of bearing fault diagnosis based on deep learning are described in the following paragraphs.

Xie [28] proposed rotating machinery fault diagnosis based on convolutional neural network and empirical mode decomposition (EMD). The EMD is used to extract the frequency domain information of the signal, and CNN is used as the classification. Guo et al. [29] proposed multi-scale continuous wavelet transform combined with CNN for bearing fault detection, but they only used CNN for classification without fully utilizing CNN’s powerful feature extraction capability. Ince [30] proposed to combine feature extraction and classification tasks into a network. Abdeljaber [31] used a one-dimensional convolutional neural network for the vibration detection of steel frames, and directly used the powerful learning ability of one-dimensional convolutional neural networks to classify faults.

Pan [32] believed that the above works used the same load to collect signals for training and testing CNN, which limits the further application of the model. Therefore, the LiftingNet deep learning network is proposed to train and test signals with different sampling frequencies and different loads. However, this method normalizes the amplitude, which leads the model not to recognize the severity of the fault.

Zhang [23] proposed WDCNN, which is an end-to-end diagnostic method for the case of variable loads. However, this method is only a single model, which has certain limitations and is more susceptible to random initialization. In addition, they performed a min-max regularization operation on the input, which made the signal distribution susceptible to individual extreme values, resulting in excessive distribution differences under different loads. Li [33] proposed to treat the original signal as a two-dimensional frequency domain signal as an input and use a multi-sensor classifier to integrate fault diagnosis. It can well overcome the limitations of a single model, but the original signal is a one-dimensional time domain signal, and transforming it into a two-dimensional signal may destroy some spatial information.

Therefore, we propose a one-dimensional fusion neural network based on D-S evidence theory, using the original signal without min-max regularization as input, and directly using the one-dimensional convolutional neural network to adaptively extract and classify the original time domain signal, and adopt it in the network. Moreover, RMSprop optimization and BN are used to suppress over-fitting of the network.

## 3. Proposed OFNN for Bearing Fault Diagnosis

**Figure 1:**
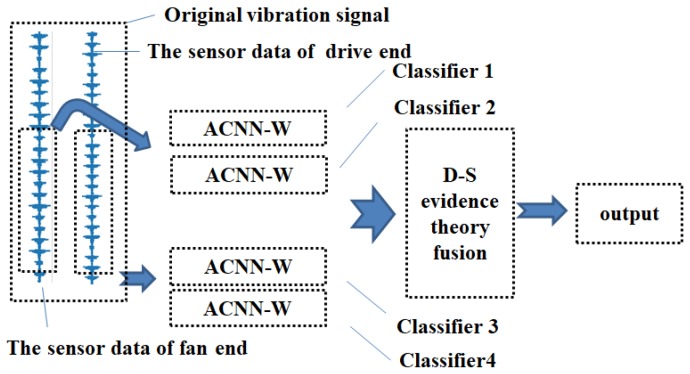
The framework of OFNN.

We propose the Adaptive one-dimensional Convolution Neural Networks with Wide Kernel (ACNN-W) as the basis for the One-dimensional Fusion Neural Network (OFNN). As shown in Figure 1, ACNN-W is proposed to learn features adaptively from raw mechanical data without prior knowledge. And we use ACNN-W as sub-classifiers of OFNN, combined with D-S evidence theory for evidence fusion. In this Section, we introduce the design of ACNN-W’s framework and the application of D-S evidence theory in one-dimensional fusion neural networks.

### 3.1. Architecture Design for ACNN-W

**Figure 2:**
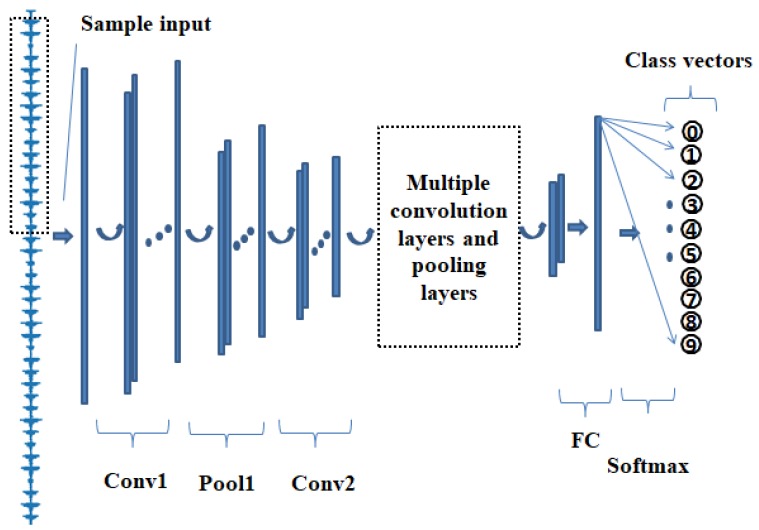
Schematic diagram of ACNN-W.

CNN consists of three layers [34], which are convolutional layers, pooling layers and fully connected layers. As shown in Figure 2, the first few layers of a typical ACNN-W consist of a combination of two types of layers—convolutional layers, followed by pooling layers—and the last layer is a fully-connected layer. Next, we will describe them in more detail.

The structural parameters of ACNN-W are shown in Table 1. The one-dimensional convolutional neural network extracts feature information from the first eight layers. The first nine layers use the ReLU function as the activation function, and the Softmax layer behind the network converts the output of the neural network into a probability distribution. Compared with the WDCNN [23], the ACNN-W network has fewer layers, requires less network parameters and has a stronger ability to avoid over-fitting.

In order to effectively suppress the neural network over-fitting and improve the ability to express, we use the Dropout algorithm in the fully connected layer. The algorithm turns the value of a layer of neurons to 0 with a certain probability, thereby preventing co-adaptation between neurons. Similarly, Zhang [23] proposed that when the first layer of the one-dimensional convolutional network is a large convolution kernel, a larger receptive field can be obtained, and the useful features for diagnosis can be learned autonomously and the over-fitting can be suppressed, so the network we propose uses a large convolution kernel at the first level.

The convolutional layer consists of a number of 1-D filters with weighting parameters. These filters combine with the input data and get an output called feature map, and each filter shares the same weighting parameters for all slices of input data to reduce training time and model complexity.

In order to improve the training efficiency of the network, reduce the internal covariate transfer, and enhance the generalization ability of the neural network. We used Batch Normalization (BN), which can accelerate deep network training by reducing internal covariate shift, reduce the fluctuation and improve the recognition rate [35].

Commonly used neural network activation functions are the sigmoid function (Sigmoid), the hyperbolic tangent function (Tanh), and the rectified linear unit (ReLU). When the absolute value of the input value is relatively large, the derivative values of the Sigmoid and Tanh functions are close to 0, which causes the error value to be propagated downward when updating the weight with the error back propagation, and the vanishing gradient problem during training the underlying network. Conversely, when the input value of the ReLU function is greater than 0, its reciprocal value is 1, which can well overcome the vanishing gradient problem.

#### 3.1.1. The Cost Function of the ACNN-W

Two cost functions have been developed to measure the error between the input vector and the target vector, which are the traditional mean square error cost function and the newly developed cross entropy cost function. Compared with the mean square error cost function, the cross entropy cost function exhibits faster convergence speed and stronger global optimization ability. So cross entropy is a widely used loss function that characterizes the distance between two probability distributions. The smaller the cross entropy, the closer the two probability distributions are. Given two probability distributions *p* and *q*, we can use *q* to denote the cross entropy of *p* as:

(1) $$H(p,q)=-\sum_{x}p(x)\log q(x)$$

where the probability distribution function satisfies:

(2) $$\forall x\;p(X=x)\in \left[0,1\right]\;\mathrm{and}\;\sum_{x}p(X=x)=1$$

Combined with mini-batch, the loss function cross entropy can be expressed as:

(3) $$L=-\frac{1}{m}\sum_{k=1}^{m}\sum_{j}p_{k}^{j}\log q_{k}^{j}$$

where *m* is the mini-batch size, *q* is the actual output value of the Softmax layer, and *p* is its target distribution [36].

#### 3.1.2. The Optimizer of the ACNN-W

The optimizer plays an important role in the training speed and classification accuracy of the neural network model. The commonly used optimizer has three kinds of optimizers: RMSprop, Adam and Adadelta. Qu [21] demonstrates the superiority of the RMSProp optimizer in bearing fault diagnosis. The optimizer can effectively prevent the premature convergence problem in the deep learning process by adaptively retaining the learning rate based on the mean value of the nearest magnitude of the weight gradient, and is suitable for processing non-stationary data such as a vibration signal. So we use the RMSprop optimizer, and the RMSprop optimizer training steps are as follows:
**Input:** Global learning rate ε, decay rate p, Initial parameter θ, Constant σ is standing at 10^−6^ (for stable values)Initialize cumulative variables r=0
**While** not reach the stop criterion **do**    Take a small batch of m samples $\left\{x_{1},x_{2},\cdots x_{m}\right\}$ from the training set and the corresponding label is $\left\{y_{1},y_{2},\cdots y_{m}\right\}$
    Gradient calculation: $g\leftarrow \frac{1}{m}\nabla_{\theta }\sum_{i}L\left(f\left(x_{i};\theta \right),y_{i}\right)$
    Cumulative square gradient: r←ρr+(1−ρ)g⊙g
    Update parameter: $\Delta \theta =-\frac{\varepsilon }{\sqrt{\delta +r}}⊙g$ ($\frac{1}{\sqrt{\delta +r}}$ Element-by-element application)    Application update: θ←θ+∇θ
**End while**

### 3.2. The Application of D-S Evidence Theory in OFNN

To reduce the impact of random initialization of the network, we use at least two ACNN-W networks to train the same data set. Therefore, as shown in Figure 1, the proposed method constructs four sub-classifiers based on ACNN-W. The vibration data measured by the two sensors in the same period is cut and used as the input of the classifiers 1, 2 and the classifiers 3, 4, respectively. In addition, the classifiers 1, 2, 3, and 4 use random initialization operations and are trained individually. After the training is completed, the vibration signals that need to be verified are respectively input into the trained network, and the class vectors of the Softmax layers of the four networks are taken out for D-S evidence fusion, thereby obtaining the final diagnosis result.

D-S evidence theory is mainly used to deal with uncertainty reasoning, which was proposed by Harvard mathematician Dempster and his student Shafer. In the currently used combination strategy, voting is simple and convenient, and has been widely applied to different ensemble learning methods. However, the main disadvantage of voting majority consent rules is that all individual models have the same weight and cannot fully exploit hidden information. The D-S evidence theory can be considered as a general extension of Bayesian theory, which can effectively deal with incomplete data. D-S evidence theory is not the probability of calculating propositions, but the probability of supporting evidence to support propositions and provides an alternative method for dealing with uncertainty inference based on incomplete information, solving the prior probability problem by tracking explicit probability measures that may lack information [37,38,39].

We use the fault type as the identification framework of D-S evidence theory *Θ* = {*A*_1_, *A*_2_, …, *An*}, and the probability value of the Softmax layer output of the sub-classifiers can well satisfy the condition of the basic probability assignment function (BPA) *m*:

m(ϕ)=0

(4) 0≤m(A)≤1,∀A⊂Θ

$$\sum_{A\subset \Theta }m\left(A\right)=1$$

where proposition *A* is a non-zero subset that identifies the frame, the symbol *m* is a measure of the subset of *Θ* and *m(A)* represents the degree of trust in *A*.

For *A* ⊂ *Θ*, in the recognition framework, the Dempster synthesis rules for the finite basic probability distribution functions *m*_1_, *m*_2_, …, *m_n_* are as follows:

(5) $$\left(m_{1}⊕m_{2}\cdots ⊕m_{n}\right)\left(A\right)=\frac{1}{1-k}\sum_{A_{1}\cap A_{2}\cdots \cap A_{n}=A}m_{1}\left(A_{1}\right)\cdot m_{2}\left(A_{2}\right)\cdots m_{n}\left(A_{n}\right)$$

(6) $$k=\sum_{A_{1}\cap A_{2}\cdots \cap A_{n}=\phi }m_{1}\left(A_{1}\right)\cdot m_{2}\left(A_{2}\right)\cdots m_{n}\left(A_{n}\right)=1-\sum_{A_{1}\cap A_{2}\cdots \cap A_{n}\neq \phi }m_{1}\left(A_{1}\right)\cdot m_{2}\left(A_{2}\right)\cdots m_{n}\left(A_{n}\right)$$

where *k* represents the degree of conflict evidence, and the coefficient 1/(1 − *k*) is called the normalization factor, ensuring that the sum of the probabilities of *BPA* is 1.

According to the Dempster synthesis rule, we can fuse the class vectors output of the softmax layer of each ACNN-W, and use the category with the highest probability value of output as the final diagnostic category.

## 4. Experiment and Network Settings

### 4.1. Enhancement and Division of Data Sets

**Figure 3:**
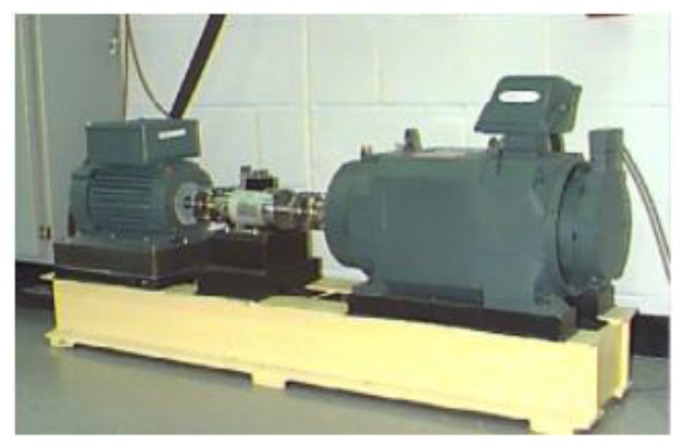
CWRU data sampling system used by CWRU.

We used a test database from the Case Western Reserve University (CWRU) bearing database with a sampling frequency of 12 kHz for verification experiments. The experiment used a 1.49 kW Reliance Electric motor, whose bearings were replaceable, and the bearing type used in this drive end is 6205-2RS JEM SKF. Figure 3 shows the data sampling system. The bearings used are processed by electrical discharge machining (EDM). In the inner raceway, the rolling elements and the outer raceway introduce faults ranging from 0.1778 mm in diameter to 1.016 mm in diameter, respectively. The faulty bearing was then reinstalled into the test motor and the vibration data of the motor load from 0.75 to 2.24 kW was recorded [40].

**Figure 4:**
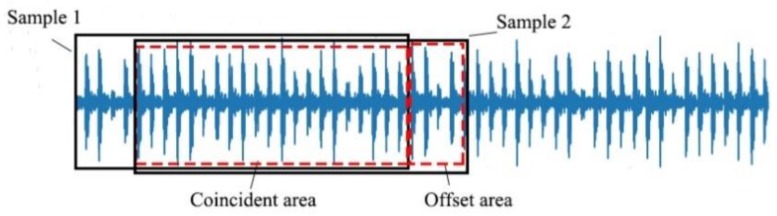
Schematic diagram of resampling sample extraction.

Data set enhancement techniques are commonly used in the field of computer vision to increase the generalization of the network by adding training samples. In view of the fact that the vibration signal collected by the acceleration sensor is a one-dimensional signal, overlap sampling is used in this paper. As shown in Figure 4, we take 2048 data points of the continuous vibration signal as a sample, and offset it by a set amount to be the second sample.

As shown in Table 2, in this paper, the shock signals of 0.75 kW, 1.49 kW, and 2.24 kW are respectively corresponding to three large data sets of A, B, and C. Each large data set contains a training set and a test set. Ten states under 0.75 kW, 1.49 kW, and 2.24 kW were numbered 1 to 10, and each state was divided by an offset of 20 to make 5000 samples. Each state of the same horsepower is randomly selected from 500 samples into the test set, and the rest is placed in the training set. Each sample consists of two parts, the drive sensor signal and the fan end sensor signal at the same time.

### 4.2. Evaluation and Settings of ACNN-W

Since the OFCNN model is based on ACNN-W, we need to evaluate ACNN-W first. In the next, we evaluate the ACNN-W network using the driver signal as an example.

#### 4.2.1. Settings of the ACNN-W

We experimented with the Tensorflow deep learning framework, using a computer configured with CPU i3-3120M and 12 GB of memory, with a selected mini-batch size of 256.

**Figure 5:**
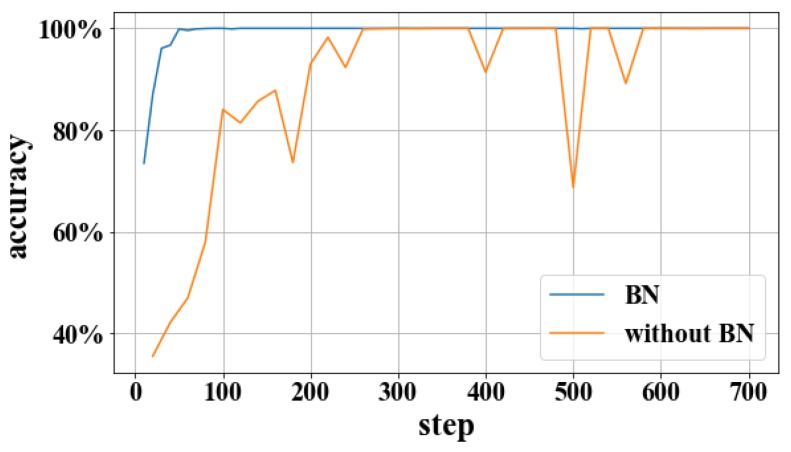
The accuracy of without BN and BN.

**Figure 6:**
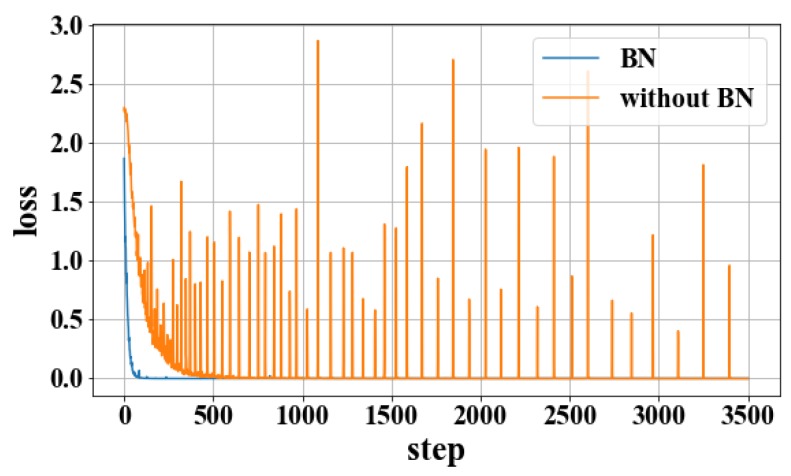
Loss function curve for without BN and BN.

In order to observe the influence of the BN layer on the model, as shown in Figure 5 and Figure 6, experiments with the BN layer were performed using the training set C and the test set C. It can be seen that the BN layer can accelerate the steady decline of the loss function and the steady increase of the recognition rate, and reduce the fluctuation of the recognition rate.

Considering the effect of different learning rate settings on the actual convergence rate, different learning rates are used for model training. Each group of optimizers and learning rates are tested 10 times, two epochs are trained each time. The average recognition rate of training set C and test set A was used as an evaluation index, and the experimental results are shown in Table 3:

It can be seen from Table 3 that the Adadelta optimizer has a poor classification result when the learning rate is low, and has a high classification result when the learning rate is large. The experimental results of the RMSprop and Adam optimizers are the opposite. After comparing the best classification results of each optimizer horizontally, we found that the RMSprop optimizer can achieve the best classification result by taking the highest recognition rate of the training set and the test set at the learning rate of 0.01. Therefore, in this paper, we select the RMSprop optimizer and set the optimization rate to 0.01.

**Figure 7:**
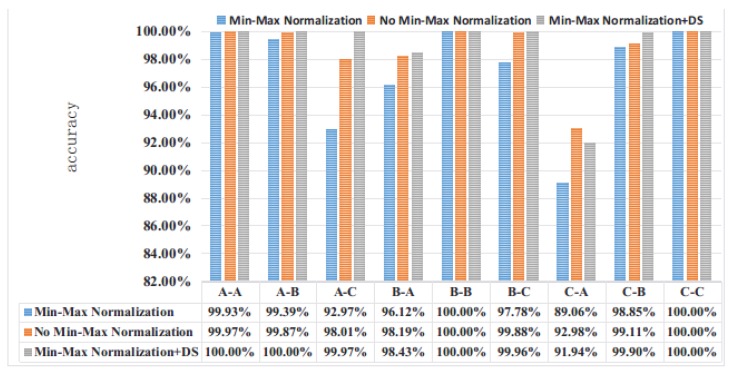
Comparison of Min-Max Normalization over 20 experiments.

For the experimental data set, we believe that if the input data is processed by Min-Max Normalization, a very small number of mutation values will affect the overall data distribution. Therefore, we will enter whether to perform Min-Max Normalization processing for experimental comparison. In order to avoid random sampling errors, each of the following experiments was repeated 20 times to average. As shown in the following figure (A-B, A on the left means the training set label, and B on the right means the verification set label):

It can be seen from Figure 7 that for the CWRU bearing database, the Min-Max Normalization processing of the input is beneficial to improve the generalization ability of the network, and is more conducive to learn more accurate features of the ACNN-W network. Even the performance of the four ACNN-Ws fused by D-S evidence is not too prominent, which takes the signal collected by the drive end as input. Therefore, the proposed ACNN-W uses BN and does not use Min-Max Normalization for experiments.

#### 4.2.2. Network Visualization Assessment

CNN is often seen as a black box, and the internal operating mechanism is difficult to understand. The nonlinear dimensionality reduction algorithm, t-SNE, was proposed for visualization of neural networks [41]. Based on the principle that t-SNE can convert the Euclidean distance into a conditional probability to express the similarity between points, we can use it to project points in high-dimensional space into low-dimensional space. Here, we select C-A with a lower recognition rate for visualization to further understand the internal operation of the model.

**Figure 8:**
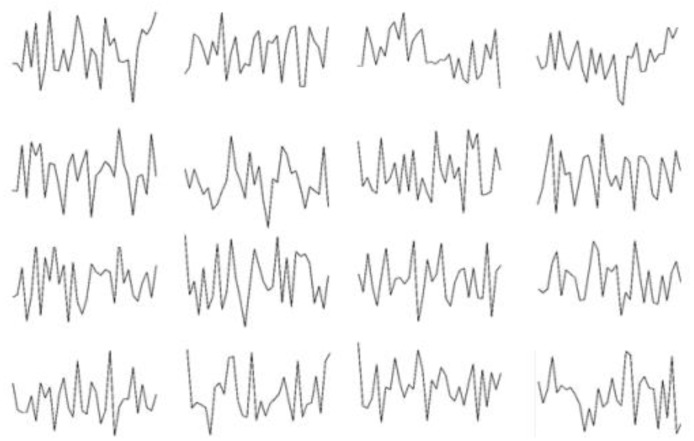
Sixteen convolution kernel visualizations of the first layer.

**Figure 9:**
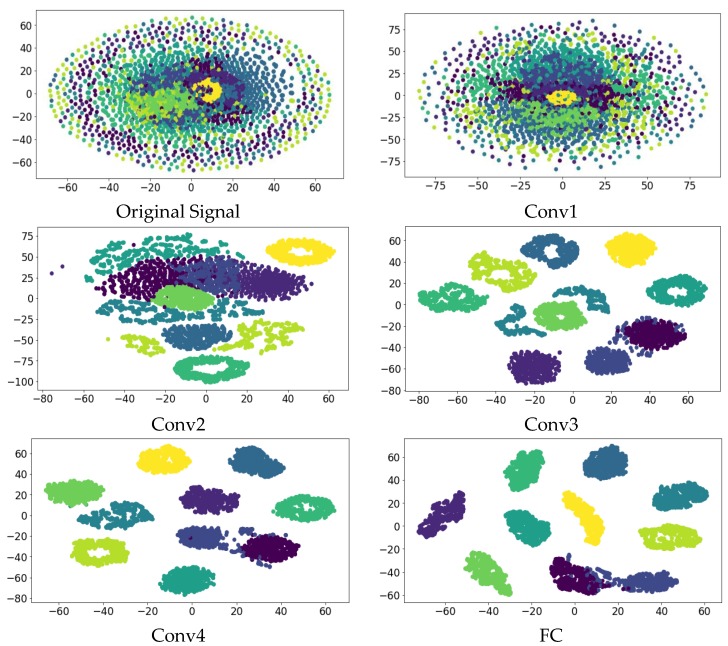
Feature visualization via t-SNE: feature representations for all test signals extracted from raw signal, four convolutional layers and the fully connected layer respectively.

Figure 8 shows the characteristics of the 16 large convolution kernels of the first layer after training the C training set. Figure 9 shows the visualization of the feature distribution of the verification set A in the four convolutional layers and the fully connected layers after training by the C training set. Using t-SNE we can find that as the layers get deeper, the features become more and more separable. It is not easily distinguishable when the original signal is input, while after feature extraction by the neural network, these features become easily divided in the fully connected layer. The results show that the low-level original signal in the input layer is converted into high-level features layer by layer, which can improve the accuracy and robustness of classification. This reflects the powerful adaptive feature extraction capability of the ACNN-W networks. In addition, we can see from the visualization of the fully connected layer that there are still some overlapping regions in the two clusters, which is consistent with our final experimental results. Therefore, the results show that our method has good feature learning ability and can learn basic fault features from the original vibration signal adaptively.

## 5. Discussion

### 5.1. Evaluation of the OFNN

**Figure 10:**
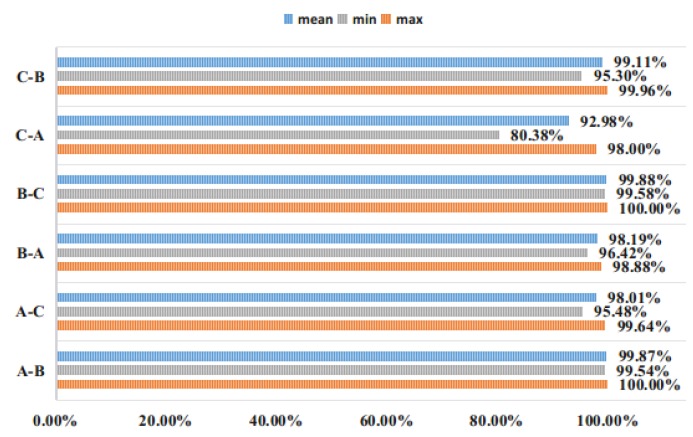
Drive end data test.

**Figure 11:**
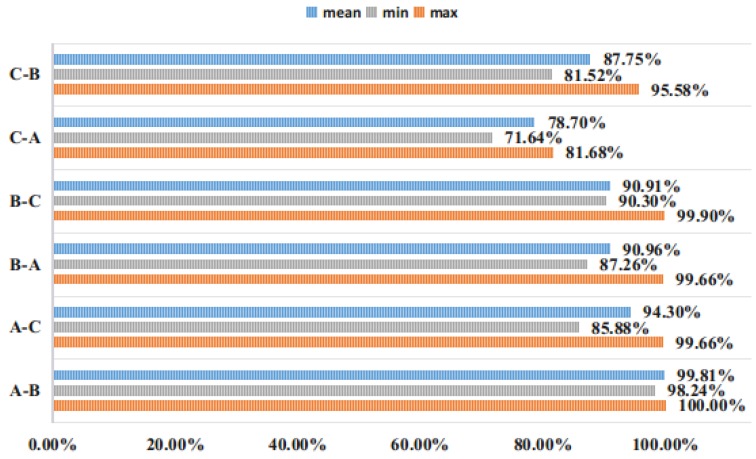
Fan end data test.

Figure 10 and Figure 11 show the ACNN-W model of the drive end sensor signal training and the ACNN-W model of the fan end sensor signal training under different loads.

To further evaluate the performance of OFNN, we used three different methods to train the OFNN model: (1) Comprehensively diagnose the test results of the four classifiers with the drive end signal as input; (2) Comprehensively diagnose the test results of the four classifiers with the fan end signal as input; (3) Comprehensive diagnosis of the CNN model trained from the signals of the two sensors.

ACNN-W-AVG-DA in Table 4 represents the average of the accuracy obtained by using only the drive end sensor signal experiment, ACNN-W-AVG-FA is the average of the accuracy obtained by the experiment using only the fan end sensor signal. OFNN-DE indicates the mean of the accuracy rate obtained by DS evidence fusion, using the drive end sensor signals. OFNN-FA indicates the mean of the accuracy rate obtained by DS evidence fusion, using the fan end sensor signals. The OFNN indicates that both the drive end sensor signals and the fan end sensor signals are used. The average accuracy of the four CNN models trained from the drive end signal is 98.66%, while the average accuracy of the model trained using the fan end signal is 92.31%. That means that the signal from the drive is more conducive to fault diagnosis than the fan end, and the multi-sensor fusion model achieves better performance than a single sensor signal training model. For most test cases, the performance of it is also higher than the performance of a single CNN model. In order to clearly observe the classification of the network, we select the most representative C-A to draw the confusion matrix.

**Figure 12:**
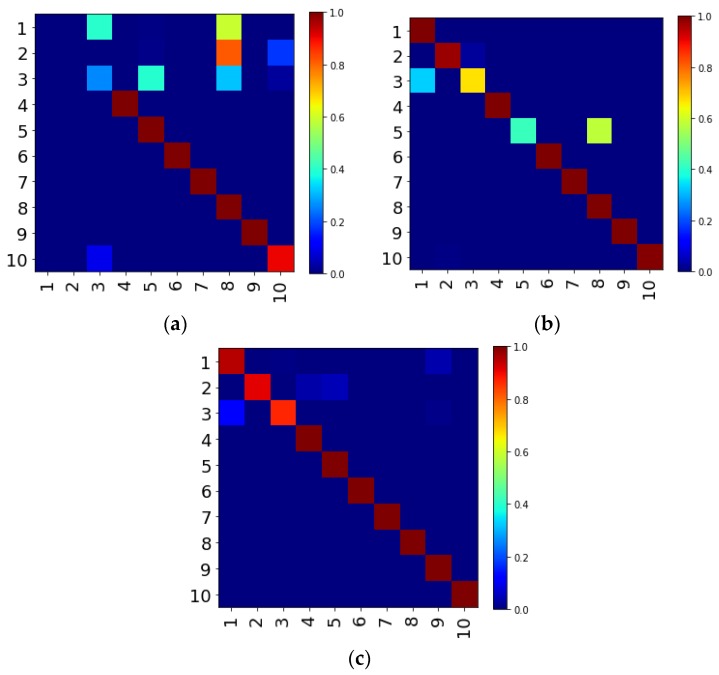
Information fusion between drive-end and fan-end predictions: (**a**) C-A fan end confusion matrix; (**b**) C-A drive end confusion matrix; (**c**) C-A confusion matrix after DS evidence fusion.

As shown in Figure 12, the vertical axis of the confusion matrix represents the actual label for each bearing state, while the horizontal axis represents the predicted label. We can find that the classification effect of the model trained by the drive end signal is better than that of the model trained by the fan end signal. The model in Figure 12a has a poor classification effect on labels 1, 2, 3, and 10. Correspondingly, the model in Figure 12b is mainly that the classification effects of labels 3 and 5 are poor. Although the recognition rate of the third type of labels is relatively low, the two models still have complementary functions in other fault type classifications. After the fusion of evidence, it can be seen from the picture c that the recognition rate of label 3 has been greatly improved, and other types of labels can almost all be recognized. The proposed method is more accurate and stable than a single CNN.

### 5.2. Algorithm Comparison and Analysis

In order to verify the recognition performance of the proposed algorithm and the current mainstream intelligent fault diagnosis algorithm, FFT-SVM, FFT-DNN [20], WDCNN [21] and other newly proposed algorithms are compared:

As shown in the Table 5, the average accuracy of the proposed OFNN model combined with D-S evidence theory under variable load conditions is higher than 99%. In A-B, B-A, B-C and C-B, the performance of FFT-SVM, FFT-DNN, WDCNN, TICNN, Ensemble TICNN [26] and IDSCNN [33] has less variation, and the accuracy of A-C and C-A is much higher than other methods. Moreover, in conjunction with Figure 7, we can see that ACNN-W exhibits better performance than WDCNN. Therefore, the excellent performance of OFNN can be attributed to the good adaptive and generalization capabilities of the proposed ACNN-W and the excellent performance of the D-S evidence theory. In particular, the accuracy of C-A increased from 93.8% of IDSCNN to 97.0%. This shows that one-dimensional convolutional neural networks are more suitable for extracting spatially related information in the original sequence than two-dimensional convolutional networks in dealing with one-dimensional signals. Compared to WDCNN, we have more comprehensively retained the original input signal, better retaining the hidden features inside the sample. The proposed method relies entirely on the one-dimensional convolutional neural network with RMSprop and BN for feature extraction without excessive manual intervention. However, due to the increase in the number of integrated individual models, the proposed method requires more computation time than the individual model. With the rapid development of modern hardware technology and training algorithms, it is believed that the integrated deep learning method will be completed more efficiently [42].

## 6. Conclusions

In this paper, we propose a method of OFNN, combined Dempster-Shafer evidence theory and the proposed Adaptive One-dimensional Convolution Neural Networks with Wide Kernel, for intelligent fault diagnosis of rolling bearings. It can be effectively achieved higher accuracy under variable load conditions, compared with the FFT-SVM, FFT-DNN, WDCNN, TICNN, Ensemble TICNN and IDSCNN models. Under the premise that we assume that the evidence provided by each classifier is independent of each other, it proves that DS evidence theory can effectively synthesize the output information of multi-classifiers, and better overcomes the local optimal problem caused by random initialization of networks. The proposed method can get rid of the dependence on manual feature extraction during diagnosing experimental bearing vibration data, and give full play to the advantages of one-dimensional neural network for feature extraction of one-dimensional original vibration signals. It is more effective and more robust than the existing intelligent diagnosis methods, overcoming the limitations of individual deep learning model. The new application of combining deep learning with integrated learning is very promising. However, as the number of integrated individual models increases, so does the amount of computer resources that are occupied. It is a research direction for bearing fault diagnosis in the future how to use integrated learning for diagnosis faster and at a lower cost. We will continue to study this topic in the future, combined with the hardware implementation of neural networks.

## Figures and Tables

**Table 1:** Structures and parameters of ACNN-W.

Number	Network Layer	Core Size/Step Size	Number of Cores	Output Size (Width × Depth)	Zero-Padding
1	Conv1	32 × 1/8 × 1	16	256 × 16	YES
2	Pooling1	2 × 1/2 × 1	16	128 × 16	NO
3	Conv2	3 × 1/2 × 1	32	64 × 32	YES
4	Pooling 2	2 × 1/2 × 1	32	32 × 32	NO
5	Conv3	3 × 1/2 × 1	64	16 × 64	YES
6	Pooling 3	2 × 1/2 × 1	64	8 × 64	NO
7	Conv4	3 × 1/2 × 1	64	4 × 64	YES
8	Pooling 4	2 × 1/2 × 1	64	2 × 64	NO
9	Fully connected layer	100	1	100 × 1	
10	Softmax layer	10	1	10	

**Table 2:** Description of experimental data set (the numbers 1 to 10 of Fault Type Label represent the 10 states of the bearing, including nine fault states and one normal state).

Fault Location		Ball			Inner Race			Outer Race			None
Fault Type Label		1	2	3	4	5	6	7	8	9	10
Fault Diameter (mm)		0.1778	0.3556	0.5334	0.1778	0.3556	0.5334	0.1778	0.3556	0.5334	0
Dataset A (0.75 kW)	Train	4500	4500	4500	4500	4500	4500	4500	4500	4500	4500
	Test	500	500	500	500	500	500	500	500	500	500
Dataset B (1.49 kW)	Train	4500	4500	4500	4500	4500	4500	4500	4500	4500	4500
	Test	500	500	500	500	500	500	500	500	500	500
Dataset C (2.24 kW)	Train	4500	4500	4500	4500	4500	4500	4500	4500	4500	4500
	Test	500	500	500	500	500	500	500	500	500	500

**Table 3:** Experiments of different optimizers and learning rates.

Learning Rate	0.0001		0.001		0.01		0.1		1	
Optimizer	Train	Test	Train	Test	Train	Test	Train	Test	Train	Test
	Accuracy									
Adadelta	10.00%	10.00%	10.00%	10.00%	10.00%	10.00%	85.14%	74.40%	99.96%	91.34%
Adam	98.70%	92.23%	99.99%	92.62%	99.92%	91.87%	97.80%	85.52%	10.00%	10.00%
RMSprop	98.68%	92.11%	99.94%	92.21%	99.99%	93.07%	30.12%	29.88%	10.00%	10.00%

**Table 4:** Comparison of different methods over 20 experiments.

	A-A	A-B	A-C	B-A	B-B	B-C	C-A	C-B	C-C	AVG
ACNN-W-AVG-DA	99.97%	99.87%	98.01%	98.19%	100.00%	99.88%	92.98%	99.11%	100.00%	98.66%
ACNN-W-AVG-FA	100.00%	99.37%	85.75%	90.65%	100.00%	93.18%	76.35%	85.56%	100.00%	92.31%
OFNN-DE	100.00%	100.00%	99.62%	98.50%	100.00%	99.90%	94.37%	99.99%	100.00%	99.15%
OFNN-FA	100.00%	100.00%	90.60%	91.61%	100.00%	93.48%	77.84%	85.16%	100.00%	93.18%
OFNN	100.00%	100.00%	99.76%	99.01%	100.00%	99.99%	97.02%	100.00%	100.00%	99.53%

**Table 5:** Comparison of different methods.

	A-B	A-C	B-A	B-C	C-A	C-B	AVG
FFT-SVM	68.6%	60.0%	73.2%	67.6%	68.4%	62.0%	66.6%
FFT-DNN	82.2%	82.6%	72.3%	77.0%	76.9%	77.3%	78.1%
WDCNN	99.2%	91.0%	95.1%	91.5%	78.1%	85.1%	90.0%
TICNN	99.1%	90.7%	97.4%	98.8%	89.2%	97.6%	95.5%
Ensemble TICNN	99.5%	91.1%	97.6%	99.4%	90.2%	98.7%	96.1%
IDSCNN	100.0%	97.7%	99.4%	99.6%	93.8%	99.9%	98.4%
ACNN-W	99.8%	98.0%	98.1%	99.8%	92.9%	99.1%	97.9%
OFNN	100.0%	99.7%	99.0%	100.0%	97.0%	100.0%	99.3%
